# Supplementary figures and images for: Super-enhancer interactomes from single cells link clustering and transcription
Source: bioRxiv. 2024 May 10:2024.05.08.593251. Preprint. [Version 1] doi: 10.1101/2024.05.08.593251 (PMC11100725; doi:10.1101/2024.05.08.593251)

Supplemental Figure 1

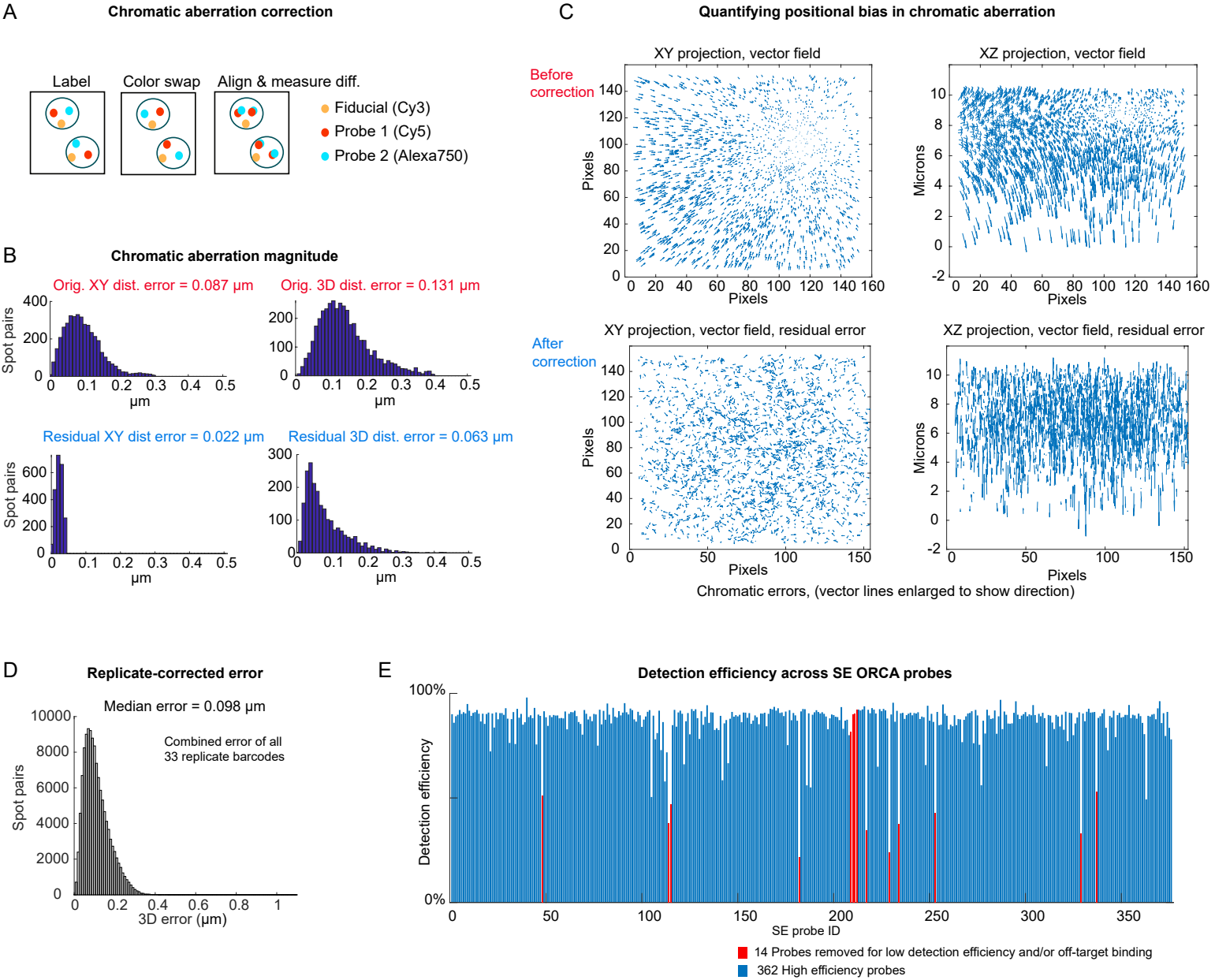

Supplement: Supplement 1 — Figure S1 ∣ Quantification of error and uncertainty, related to Figure 1. A. Schematic illustration of our approach for measuring chromatic aberrations. B. Original and corrected chromatin aberration. Note the correction is more accurate in x,y. C. Field portraits of the spatial organization of chromatic aberration. Each graph represents the field of view, each arrow starts at a position of measured chromatic aberration and points in the direction of displacement between the fluorophores. The length of the arrows has been uniformly stretched to be visible on the plot. After correction, the spatially correlated nature of the aberration has been removed. D. Quantification of measurement error by replicate labeling. Plot shows the aggregate of all replicate labels, performed up to 376 imaging rounds after the original measurement to capture the greatest extent of potential sample degradation over time. E. Detection efficiency per SE barcode. Barcodes are ordered as in Table S1. A total of 14 probes were removed for low detection efficiency and/or off target reactivity and are marked in red. [file media-1.pdf]

Supplemental Figure 2

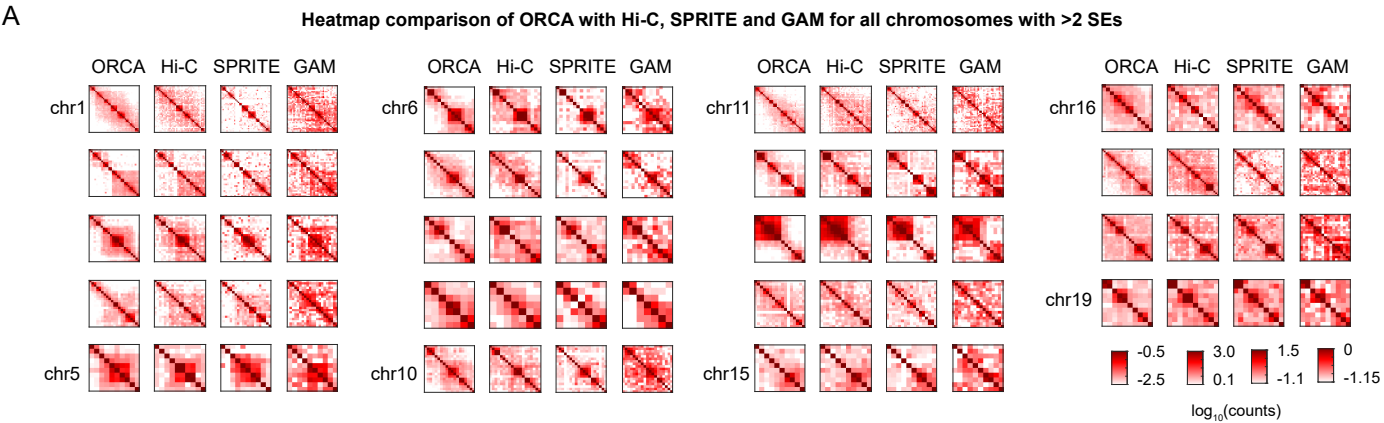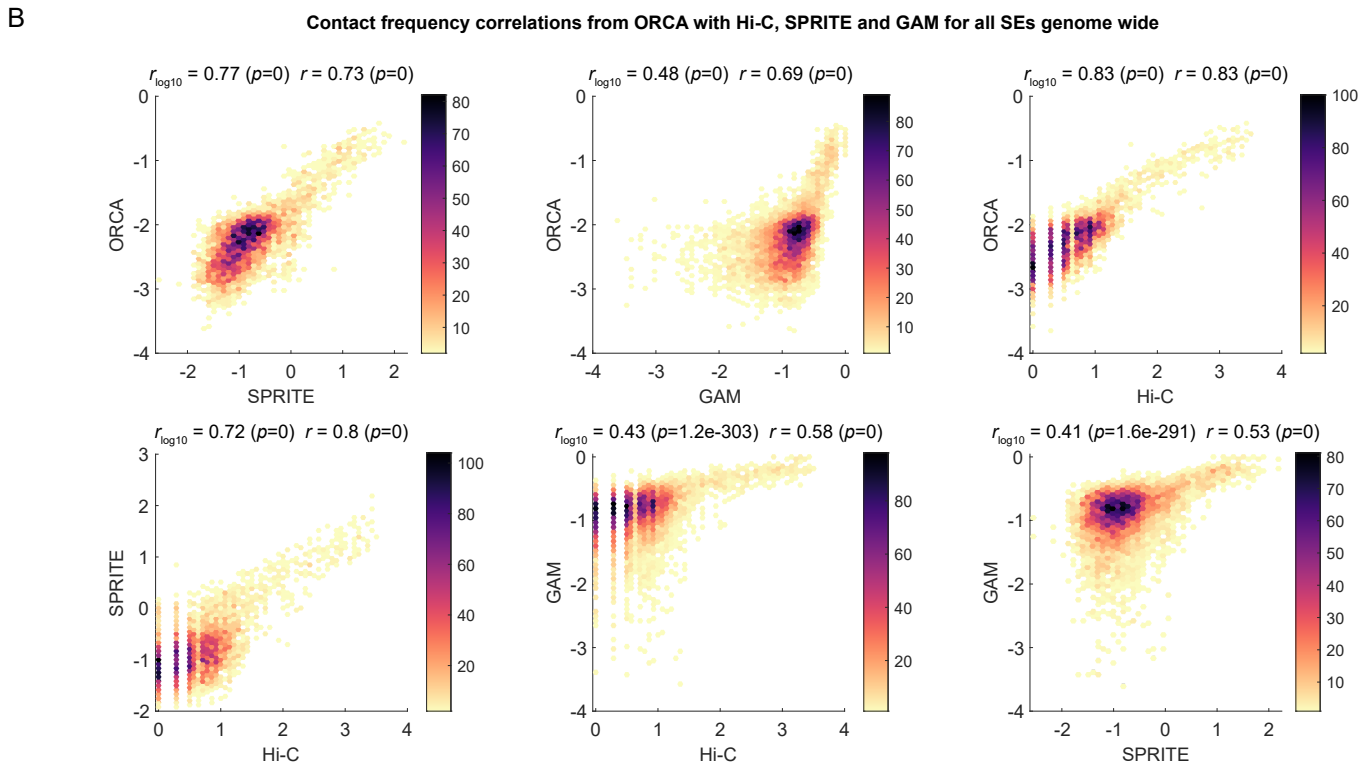

Supplement: Supplement 2 — Figure S2 ∣ Comparison of ORCA, Hi-C, SPRITE and GAM, related to Figure 1. A. Heatmaps of the contact frequency per chromosome for ORCA, Hi-C, SPRITE, and GAM. ChrX is not shown as it contains only 2 SEs. B. Correlation of the measured contact frequency between the 4 methods. [file media-2.pdf]

**Supplemental Figure 3**

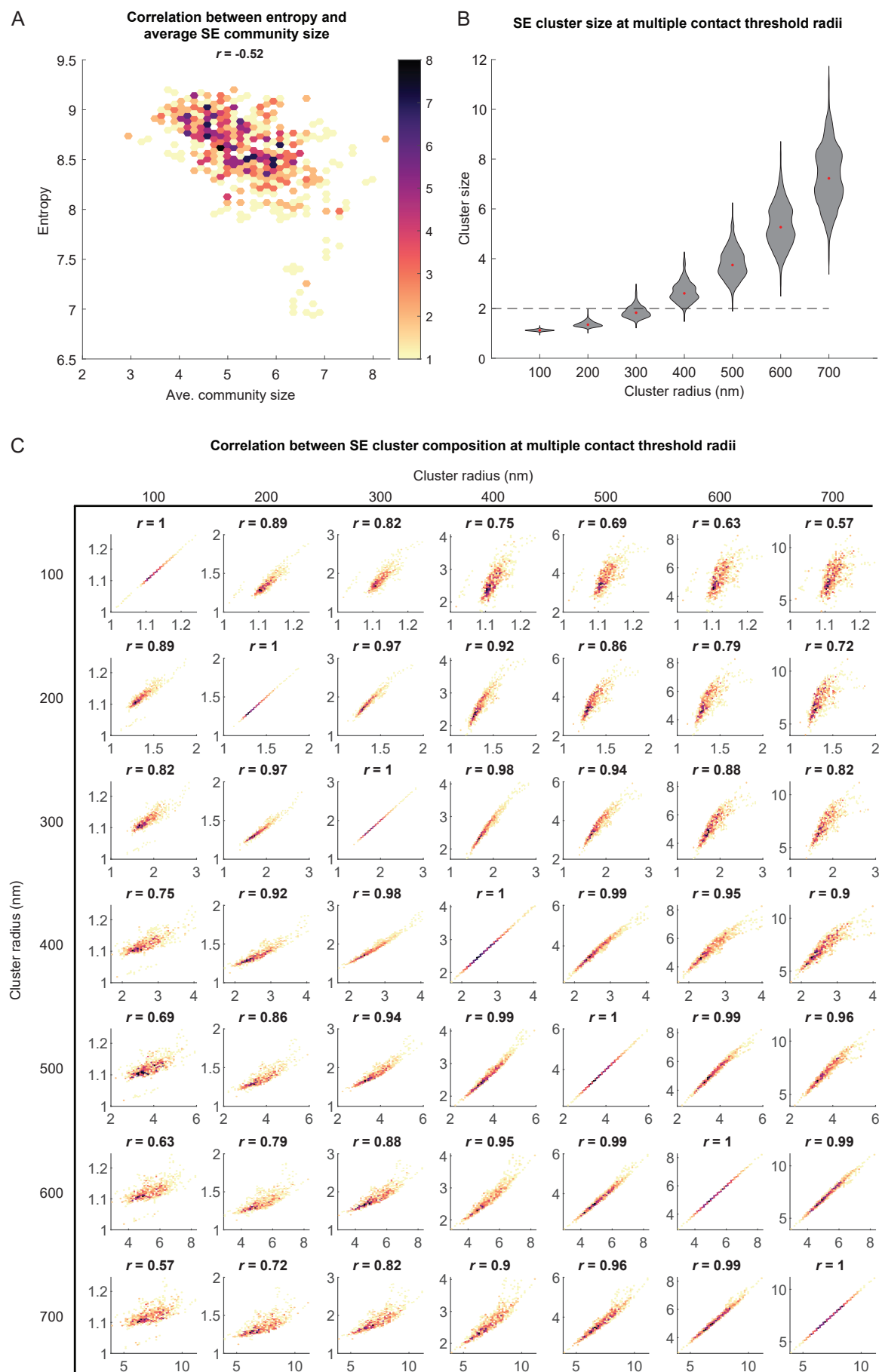

Supplement: Supplement 3 — Figure S3 ∣ Effect of community size on entropy and effect of cluster-size threshold on community size, related to Figure 3. A. Correlation of entropy and community size density of SEs indicated by the color map, Pearson’s r is shown. B. Distribution of average cluster size for each SE, shown as a violin plot, as a function of the cluster radius. Dotted line shows that at cluster-radii greater than 400 nm, most SEs are on average in a cluster, whereas at 200 nm radius all SEs are on average alone. C. Correlation plots comparing the effect of cluster radius threshold on the degree of clustering exhibited by each SE. Thresholds of 400-700 all show correlated distributions of cluster sizes with Pearson’s r greater than 0.9. [file media-3.pdf]

**Supplemental Figure 4**

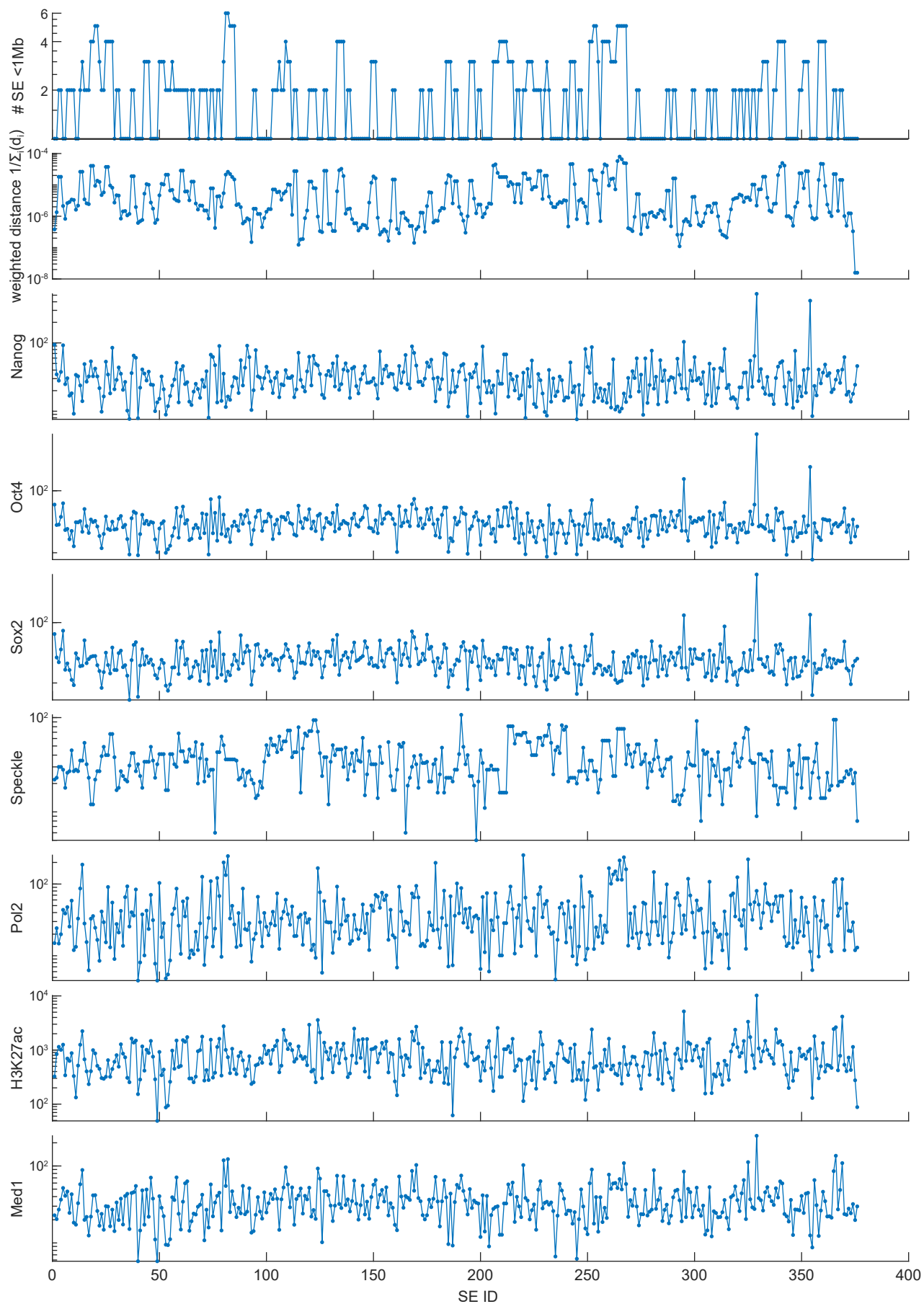

Supplement: Supplement 4 — Figure S4 ∣ Correlates of community size, related to Figure 4. Graphs show the values of the indicated factors which were used to correlate to and predict community size. The x-axes (SE ID) are aligned for comparison between correlates, and are sorted by genome location as in Figure 1. [file media-4.pdf]
