# Supplementary material for: Super-enhancer interactomes from single cells link clustering and transcription
Source: bioRxiv. 2024 May 10:2024.05.08.593251. Preprint. [Version 1] doi: 10.1101/2024.05.08.593251 (PMC11100725; doi:10.1101/2024.05.08.593251)

Supplemental Figure 5

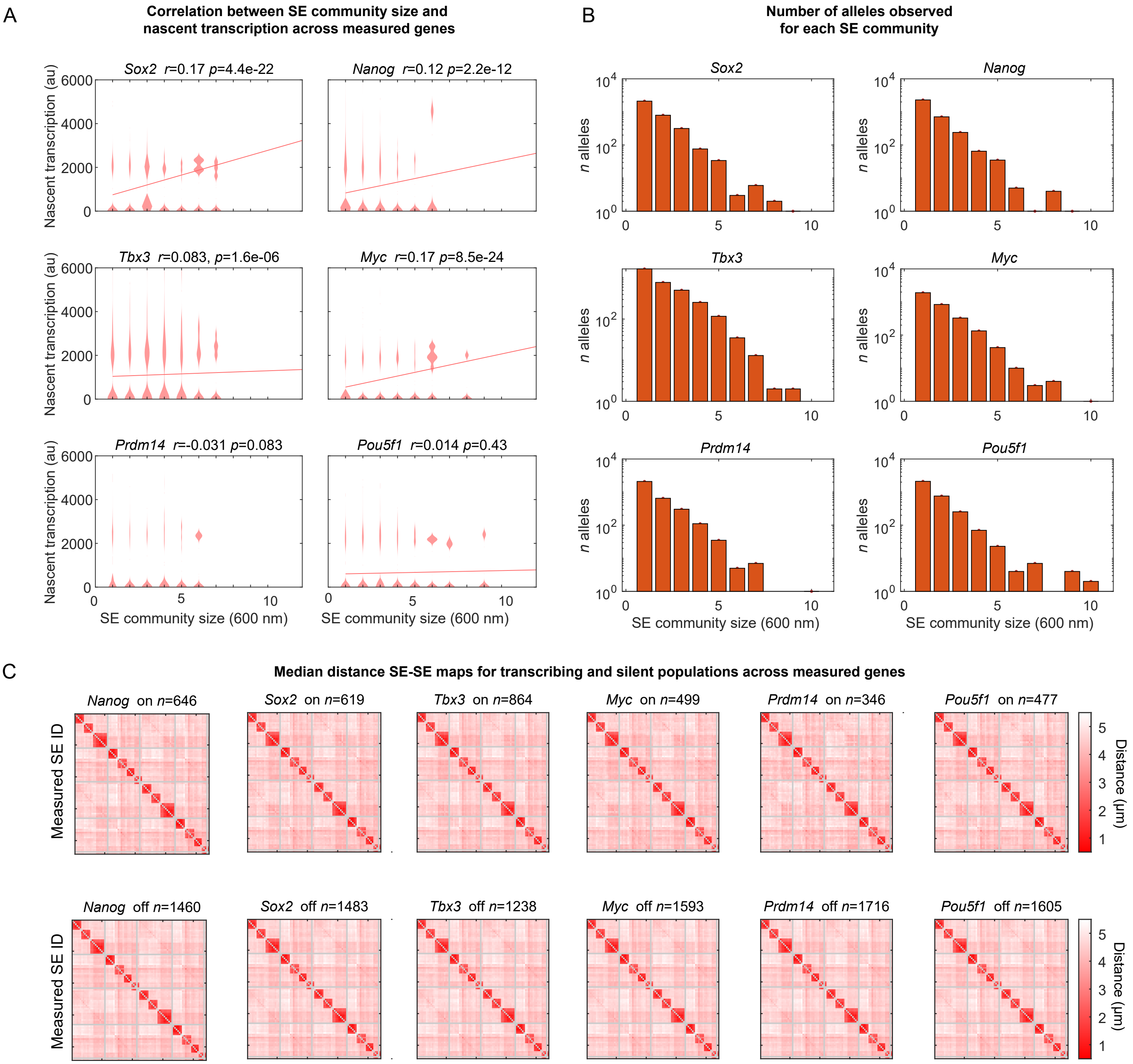

Supplement: Supplement 5 — Figure S5 ∣ Relation between transcription activity and enhancer communities, related to Figure 5. A. Distributions of nascent transcription intensity for Nanog for different community sizes. Pearson’s correlation coefficient and p-values are indicated on the plot and the line over the violins is the best linear regression through all the data. B. Number of alleles (number of cells x 2 chromosomes in each cell) for each community size. C. Median pairwise distance maps for nascent, transcriptionally ‘on’ and ‘off’ alleles for each of the 6 measured genes. [file media-5.pdf]
